# Electrophoretic Concentration and Electrical Lysis of Bacteria in a Microfluidic Device Using a Nanoporous Membrane

**DOI:** 10.3390/mi8020045

**Published:** 2017-02-03

**Authors:** Md. Shehadul Islam, Ali Shahid, Kacper Kuryllo, Yingfu Li, M. Jamal Deen, P. Ravi Selvaganapathy

**Affiliations:** 1 Department of Mechanical Engineering, McMaster University, Hamilton, ON L8S 4L7, Canada; shehadul.islam@gmail.com (M.S.I.); shahia@mcmaster.ca (A.S.); 2 Department of Biochemistry and Biomedical Sciences, McMaster University, Hamilton, ON L8S 4L7, Canada; kacper.kuryllo@thalmic.com (K.K.); liying@mcmaster.ca (Y.L.); 3 Department of Electrical and Computer Engineering, McMaster University, ON L8S 4L7, Canada; jamal@mcmaster.ca

**Keywords:** microfluidic, sample preparation, electrical lysis, *Escherichia* (*E.*) *coli*, polycarbonate (PC) membrane, nanoporous membrane

## Abstract

Pathogenic bacteria such as *Escherichia coli* O157, *Salmonella* and *Campylobacte*r are the main causes for food and waterborne illnesses. Lysis of these bacteria is an important component of the sample preparation for molecular identification of these pathogens. The pathogenicity of these bacteria is so high that they cause illness at very low concentrations (1–10 CFU/100 mL). Hence, there is a need to develop methods to collect a small number of such bacterial cells from a large sample volume and process them in an automated reagent-free manner. An electrical method to concentrate the bacteria and lyse them has been chosen here as it is reagent free and hence more conducive for online and automated sample preparation. We use commercially available nanoporous membranes sandwiched between two microfluidic channels to create thousands of parallel nanopore traps for bacteria, electrophoretically accumulate and then lyse them. The nanopores produce a high local electric field for lysis at moderate applied voltages, which could simplify instrumentation and enables lysis of the bacteria as it approaches them under an appropriate range of electric field (>1000 V/cm). Accumulation and lysis of bacteria on the nanoporous membrane is demonstrated by using the LIVE/DEAD BacLight Bacterial Viability Kit and quantified by fluorescence intensity measurements. The efficiency of the device was determined through bacterial culture of the lysate and was found to be 90% when a potential of 300 V was applied for 3 min.

## 1. Introduction

Microbiological contamination of water is one of the major reasons for waterborne diseases. Conventional culture-based methods for its detection are time consuming and compromise the timeliness of health advisory warnings [1]. Moreover, since some pathogens remain in viable but non-culturable state, these techniques may underestimate the total number of viable bacteria [2]. Molecular biology-based approaches such as identifying unique stretches of DNA that are specific to particular pathogen and analyzing its presence in water sample are accurate, extremely sensitive and can be performed rapidly [3,4] appropriate sample preparation steps are needed before analyzing these biomarker [4,5,6]. In the case of environmental samples such as water, a large volume of sample (500 mL) has to be concentrated, the bacterial contents lysed and the DNA and other biomarkers that are embedded in the cells extracted for analysis. Recently, microfluidic lab-on-chip technology has been used for sample preparation and detection of biomarkers such as DNA and proteins for medical diagnostics in an automated fashion from small samples [7,8,9,10,11].

An important step in sample pre-treatment is cell lysis. Conventionally, in the laboratory, chemical and mechanical methods are used to lyse the cell [12]. Recently, a number of microfluidic devices have been developed that lyse cells using chemical, thermal, mechanical, electrical and electrochemical methods [13,14,15,16,17,18,19,20,21,22,23,24]. The chemical method is the most widely used technique and it has well established protocol [13]. In this method, the sample is mixed with various lytic agents such as sodium dodecyl sulphate (SDS), triton X-100 or proteinase K [14]. The lytic agents interact with phospholipids on the membrane surface which results in pore formation. Alternatively, deionized (DI) water has been mixed with the sample to generate an osmotic pressure difference across the cell membrane which then leads to cell lysis [15]. Although chemical methods are widely used for cell lysis, they require reagents and additional unit operation [25,26,27,28] such as mixing, and are more suited for laboratory rather than field settings. In addition, the chemical reagents used in lysis may interfere with downstream processes such as amplification and therefore necessitate an additional DNA extraction step [29].

Thermal methods are also used for call lysis in micro fabricated devices [22,30]. Exposure to elevated temperatures (>100 °C) for short durations of time ruptures the cell membrane. PCB microfluidic device with integrated resistive heaters has been recently used to lyse cell by using localized heating [31]. However, thermal methods are not suitable for large samples that contain low concentrations of bacteria [16,17] as their efficiency is very low. Alternatively, cells can be lysed by mechanical impingement on nanoscale barbs as they are flowed through the device. Though this method is very efficient, it has some drawbacks. Cell debris produced by this method consists of very small fragments which are very hard to separate from DNA [16]. In addition, the fabrication of the nanoscale mechanical barbs is difficult and costly. Recently, cells have been lysed electrochemically by local hydroxide ion generation [20,21,32]. In an alkali environment, excess OH^−^ ions cleave the fatty acid-glycerol bond in phospholipid molecules, resulting in lysis. Again, this requires a constant current flow into the sample to generate the appropriate pH and is therefore energy intensive. Furthermore, this method does not separate the DNA from the rest of the cell debris that could potentially contain inhibitors for downstream processes. It could also lead to irreversible denaturation of the target DNA. In addition this method also requires some degree of photolithographic microfabrication to form the microelectrodes that generate OH^−^ ions making it expensive. A variation of this method has been demonstrated in which titanium dioxide particles are used as photo catalyst to generate the OH^−^ ions upon excitation from a UV LED light source. A continuous supply of UV was needed and the time required to lyse cell was high [33].

In contrast to the above methods, the direct application of electrical pulses to a cell can also damage the cell membrane and form pores in a process known as electroporation. Electroporation was originally developed in the early 1980s, as a method to temporarily damage the cell membrane and form pores through which external genetic material could be inserted into the cell [34]. If the duration of the electric pulse is short then the membrane reforms and the cell is viable again. Long pulses or DC potentials have been applied to irreversibly damage the cell membrane and lyse the mammalian cell without the use of reagents [23,25,35,36]. One of the main advantages of this method is that it is rapid and does not need any reagents. Electrical lysis requires high electric field (critical electric field 1–2 kV/cm [18,37] that have been obtained in microfluidic devices by application of a high voltage or a reduction the distance between electrodes through microfabrication [37,38,39]. However, micro fabricated electrodes are expensive and necessitate the use of alternating current (AC) and pulsed electric fields to avoid bubble generation and clogging [40,41]. Recently, direct current (DC) voltages have been used to lyse bacteria [42] and red blood cells [43] in a continuous flow through manner. Although promising, these designs consume significant power and do not concentrate the lysed DNA into a small volume which is important for environmental applications.

In this paper, we develop a continuous flow device that is capable of electrophoretically pre-concentrating bacteria from a sample into a small volume and then lyse it using a localized high electric field without the use of micro fabricated electrodes or lysis reagents. We show that this device avoids the typical problems associated with electrical cell lysis such as Joule heating and bubble generation due to its continuous flow operation and the use of lower voltages. We also demonstrate that this method separates the DNA from the cellular contents and thus makes it suitable for amplification. The throughput of the device can be increased by either increasing the cross-sectional area of the membrane interface or by parallelizing the format. This device and its form factor is capable of cell lysis from various matrices in a reagent free and simple manner and has significant potential in compact lab-on-chip devices.

## 2. Electrical Cell Lysis

Cells can be lysed when exposed to an appropriate external electric field. When such an electric field is applied, the cell membrane acts as a capacitor and a potential difference is established between intercellular and extracellular regions. This difference is known as the transmembrane potential (TMP) and for a spherical cell can be determined from [44]:

TMP: ∇∅(*t*) = *FrE*cosα (1 − exp(−*t*/*t*_m_)) (1)

where *r* is the radius of the cell, *E* is the external electric field, *t*_m_ is the charging time of the membrane, *F* is the cell shape factor and α is the angle between the field line and the normal at the point of interest in the membrane surface.

For many of the cells of interest, the charging time *t*_m_ is on the order of several hundred nano seconds. Hence, for a DC voltage *t* is much higher than *t*_m_. Therefore, we can write Equation (1) as:

∇∅(*t*) ≈ 1.5*rE*cosα (2)

where *F* is 1.5 for a spherical cell. For an elongated cell, Equation (1) can be written as:

∇∅(*t*) ≈ 0.5*LE*cosα (3)

where *F* is 0.5 for an elongated cell and *L* is the characteristic long dimension of the cell and the cell is parallel to *E* [45,46].

When this potential is about 0.2–1 V, the cell membrane becomes permeable as small pores are created on its surface [19]. This process is called electroporation. The mechanics of electroporation is not entirely understood. However, the most accepted and widely used model for electroporation based on the electromechanical compression of the cell membrane, as proposed by Zimmermann [47], is that the attraction of opposite charges induced on the inner and the outer membrane generates a compressive pressure which makes the membrane thinner and permeable to the medium.

Depending on the field strength and duration of the field, these pores might be transient or permanent. If the field strength is low and applied only for a short period of time, then the cell can re-seal itself and this process is known as reversible electroporation. Reversible electroporation is usually used to insert external material such as DNA, RNAi, small molecules or quantum dots into the cells. Irreversible pores can be created by increasing the intensity and exposure to the electric field. This process is known as irreversible electroporation and often results in cell lysis. Initially, the pores formed are small and the cytoplasmic macromolecules are retained inside the cell. However, small ions and water are permeable through those pores. In order to maintain the osmotic balance, water permeates in, the cell swells up, and eventually, cell membrane rupture [48]. Depending on the size and type of the cells, an electric field strength in the range of 600 to 2000 V/cm is required to lyse them [49].

## 3. Device Design

**Figure 1:**
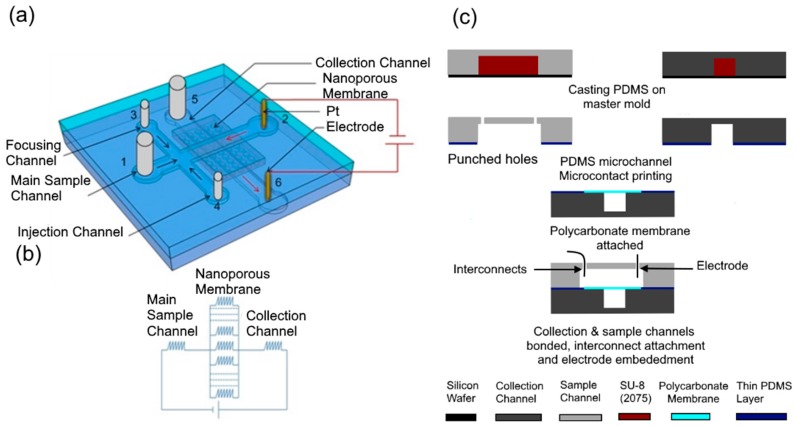
(**a**) Schematic design of the microfluidic cell lysis device. It consists of two microfluidic layers that are separated by a nanoporous membrane at their intersection. The red arrows indicate the direction of the electric field. The black arrow in the microchannel indicates the direction of flow of the sample and the buffer; (**b**) equivalent electrical circuit of the device; (**c**) fabrication sequence of microfluidic device.

Electrical lysis of the cell depends on the electric field in the vicinity of the cell as well as the duration of exposure to the electric field. Our device consists of a nanoporous membrane sandwiched between two microchannels, sample (8 mm × 80 µm × 50 µm) and collection (8 mm × 80 µm × 50 µm) channels with electrodes embedded in their reservoirs (Figure 1a). Application of a potential difference between the electrodes in the reservoirs of the sample and collection channels produces a high electric field across the nanopores at the intersection of two channels while the electric field in the microchannels remains low as the nanopores offer high resistance compared to the microchannels. Two side channels, namely the injection and focusing channels, attached to the main sample channel, are used in characterization experiments to inject a controlled volume of sample into the device. Under normal operation, the sample (bacteria) is flowed in the main sample channel from the injection channel inlet (port 4) and a buffer solution is flowed from the main sample channel inlet (port 1) and focusing channel inlet (port 3) to the outlet (port 2) where it flows out of the device through a fluidic interconnect. The buffer solution is also flowed from the collection channel inlet (port 5) to its reservoir while the electric field is applied between the electrodes in the reservoirs. The operation is performed in two steps. In the first step, flow velocity and the applied voltage are optimized to accumulate the bacterial population present in the sample. In the second step, the accumulated bacteria are electrically lysed and the DNA extracted into the collection channel.

The cells that flow through the main sample channel from the injection channel encounter four kinds of forces namely: drag force due to the pressure driven (*F*_P_) and elect osmotic (*F*_eo_) flow, electrophoretic force (*F*_ep_), gravitational force (*F*_g_) on the cells.

*F*_P_ + *F*_eo_ = 6πη*r(U*_P_ + *U*_eo_*)* (4)

*F*_ep_ = 6πη*r U*_ep_ *F*_g_ = *mg* (5)

where *r* is the radius of cell, η is the viscosity of liquid, *m* is the mass of the cell and *g* is the acceleration due to gravity. *U*_P_ Pressure driven velocity, *U*_eo_ Electro osmotic velocity, *U*_ep_ Electrophoretic velocity

*U*_eo_ = μ_eo_*E* (6)

*U*_ep_ = μ_ep_*E* (7)

μ_eo_ = Electro osmotic mobility, μ_ep_ = Electrophoretic mobility.

The drag force due to the pressure driven flow in the sample channel moves the bacteria to port 2. In the accumulation step, when the polarity of the potential applied in port 2 is negative, the electrophoretic force on the bacteria is in the opposite direction of the drag force, pulling it towards the nanoporous membrane. As the electric field lines penetrate the nanoporous membrane, the electrophoretic force tends to pin the bacteria to the membrane surface while the drag force due to the flow tends to sweep it away from the membrane. The electroosmotic flow in typical operating conditions is small compared to the pressure drive flow and can be ignored. Under suitable flow rate and applied potential, the electrophoretic force can be greater than that the drag force and the bacterial population in the sample accumulates onto a confined area on the nanoporous membrane.

The device design and operating conditions can be optimized such that the entire bacterial population in the sample is accumulated on to the membrane while at the same time maintaining the localized electric field at the nanopores lower than the critical electric field for lysis of bacteria. In the lysis (second) step, the pressure driven flows from the injection and focusing channels are stopped while the voltage is increased such that the local electric field in the vicinity of the nanopores is above the critical electric field for lysis to occur. Since the resistance of the microchannel is lower than that of the nanopore, its electric field is lower. This leads to lysis of bacteria that have been accumulated on the nanoporous membrane and extraction of negatively charged DNA through the nanopore into the collection channel. Since many thousands of pores exist at the intersection between the two microchannels, the several thousands of the accumulated bacteria can be lysed.

In the design of the device, the height of all microfluidic channels was set as 50 μm. The widths of the main sample and collection channels were set as 80 μm, while those of the focusing and injection channels connected to the main sample channel were set as 40 μm. In addition, the length of the sample microchannel was set as 8 mm based on operational and characterization considerations. The distance of the reservoirs with electrode from the center of the membrane was 4 mm. These dimensions were chosen based on optimization of the electrical resistances of these channels and the membrane, as well as the fabrication constraints to ensure that a significant proportion of the overall potential drops across the membrane. For instance, if the widths of main sample and collection channels are larger, then the number of pores in the intersection will be higher and the total resistance of the membrane will be lower. This will require a higher potential to be applied for lysis. However, if the width is very small, the resistance of the main sample channel will increase, leading to cell lysis in that channel. Similarly, the distance of the intersection from the waste and collection reservoirs is another important parameter to be optimized. If this distance is longer, the resistance of the main sample channel will be higher. However, reducing the distance also reduces the time available for the electrophoretic force to counter the drag force and accumulate the bacteria on the membrane.

Polyvinylpyrrolidone (PVP) coated polycarbonate nanoporous membrane is chosen for this device. These membranes have a single pore size, are thin and easy to integrate with PDMS compared to other membranes. Polyvinylpyrrolidone (PVP) coating is used to render them hydrophilic which help the sample solution to wet the membrane and make electrical contact through the membrane. In addition, the PVP coating is suitable for minimizing the electroosmotic flow [50]. Membranes with various pore sizes are commercially available and their pore density varies with pore size. Table 1 shows the various commercially available pore sizes and corresponding pore densities and thicknesses of the membranes. Since the area of the membrane at the intersection between the sample and collection channel is 80 µm × 80 µm in this design, the voltages that must be applied to obtain a critical electric field of 1600 V/cm across the nanopores to lyse bacteria [42] can be calculated for various membrane specifications as shown in Table 1. This was calculated by calculating the resistance associated with an individual pore of a specific diameter. Then the total number of pores in an 80 µm × 80 µm area was computed by using the pore density information in Table 1. These resistances were considered to be in parallel as shown in Figure 1b to compute the electric resistance of the membrane. Then the membrane resistance was considered in series with the resistance of the channels to determine distribution of the applied voltage and compute the electric field in the pores.

From Table 1, it is apparent that the critical electric field for lysis can be generated at the nanopores of 0.08 and 0.1 μm pore size membranes by applying just 35 and 66 V, respectively. However, not all the membranes represented in Table 1 can be used in this application. Any pore size greater than 0.5 μm will not be able to retain *Escherichia* (*E.*) *coli* as its size is 0.5 μm in diameter and 2 μm in length. On the other hand, any pore size smaller than 0.2 μm poses fabrication and operational problems as priming the pores to form electrical and fluidic interconnections between the sample and the collection channel become difficult. Therefore, a 0.4 μm pore size has been selected here for use.

## 4. Materials and Methods

### 4.1. Fabrication of Device

**Figure 2:**
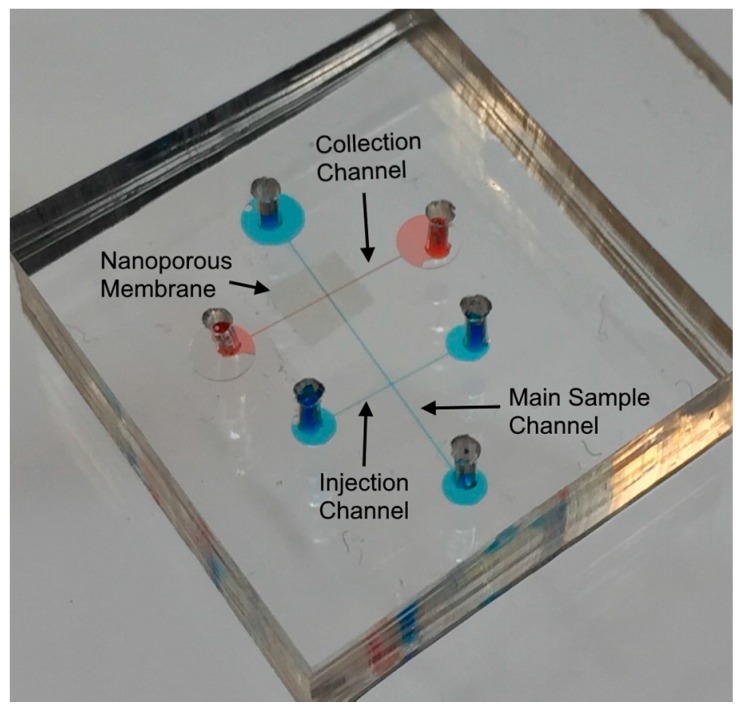
Fabricated microfluidic cell lysis device.

The fabrication process of device is shown in Figure 1c. This device consists of two layers (Figure 1a). Microchannels in the top and bottom layers were fabricated in PDMS by using a rapid prototype and soft lithography process [51]. The mask layout was designed in AutoCAD (Autodesk Inc., San Francisco, CA, USA) and printed using ultra high-resolution laser photo plotting on a transparency sheet. SU8-2075 (50 mm thick) negative photoresist (MicroChem Corporation, Westborough, MA, USA) was used to lithographically pattern a master mold of the top and bottom layers of the device. Polydimethylsiloxane (PDMS) pre-polymer mixture (Sylgard 184 kit, Dow Corning Corporation, Midland, MI, USA; 10:1 ratio of the base and cross linker) was cast on the master mold, and then cured at room temperature for 24 h. Next the PDMS replica was peeled off the master mold and cut into pieces containing individual channels. The inlet and outlet access ports were punched out at the reservoir areas for the top layer. Polycarbonate membrane (0.4 µm pore size, pore density 1 × 10^8^, Sterlitech Corporation, Kent, WA, USA) coated with polyvinylpirodyne (PVP) was purchased and was cut into a square section of 3 mm × 3 mm. In order to attach a nanoporous membrane on the microchannel, a thin layer of PDMS was obtained by spinning PDMS pre-polymer (base/curing agent: 1/3) on the silicon wafer at 8000 rpm for 2 min [52]. The microchannel top surfaces which was used for the bottom layer was placed on the thin uniform PDMS pre-polymer and lifted off. Next, the membrane was placed on the middle of the bottom layer. Then, the top surface of top layer was placed on the thin uniform PDMS pre-polymer and lifted off. The PDMS piece with membrane attached was then aligned and bonded with the top layer PDMS piece. Finally, whole device (Figure 2) was kept at room temperature for overnight (~16 h) and then cured at 120 °C for 1 h. Inlet reservoirs were connected to a thin tube where metal electrodes (Pt wire, 0.25 mm in diameter) were inserted to outlet reservoirs of both channel.

### 4.2. Experimental Setup and Procedure

**Figure 3:**
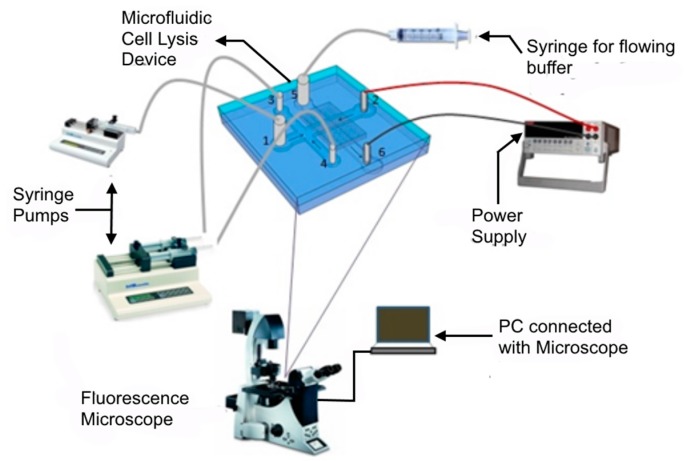
Experimental setup used for cell lysis. Apart from the chip, it consists of the fluidic system to control the fluid flow, the electrical power supply to apply the electric field and a microscope to visualize the accumulation and lysis of the cells at the nanoporous membrane.

The experimental setup (Figure 3) to study the accumulation of bacteria and lysis consists of four major parts: a microfluidic device (described in the design section), the bacteria and buffer handling system (syringe pumps, syringe, and inlet-outlet connection), power system (power supply and electrodes) and a monitoring unit (microscope and PC).

To demonstrate accumulation of cells, *E. coli* with bacteria viability kit (Syto 9 and PI) had been used. Syto 9 can permeate the intact membrane of bacterial cells, binds with DNA and forms a green-fluorescent complex. In contrast, propidium iodide (PI) cannot permeate the intact membrane, but it can permeate the damaged membrane and binds to DNA forming a red-fluorescent complex. By using an appropriate mixture of these two dyes, the intact cells can be stained to be green-fluorescent while cells with damaged membrane can be stained to be red fluorescent. Since, these dyes have been used during preparation of sample, intact bacteria fluoresce green (500–550 nm) when excited with blue light (488 nm) and lysed bacteria fluoresce red (595–660 nm) when excited with green light (561 nm).

First, the sample and the collection channels were loaded with phosphate buffer (1.35 mM KH_2_PO_4_, 2 mM Na_2_HPO_4_, 0.05% Tween20, pH 7) and platinum wires (diameter 0.25 mm) were inserted into the reservoirs 2 and 6 (Figure 3). Electrodes were connected to a power source (KEITHLEY 2410, Keithley Instruments, Cleveland, OH, USA). The membrane was first primed by applying 100 V and the current was recorded. Priming was done in order to get continuous flow of current. Then, all the trapped air bubbles were removed by flowing buffer from reservoirs 3 and 5 by a syringe. Reservoirs 3 (buffer) and 4 (bacteria) were connected to syringe pumps with the same flow rate (KD Scientific 200, KD Scientific Inc., Holliston, MA, USA) and reservoir 1 was connected with another syringe pump whose flow rate can be set independently (KD Scientific 100). Next, all pumps were started with a flow rate of 100 μL/h. A syringe was used to flow buffer in the bottom channel manually through reservoir 5. When bacteria were observed to approach the membrane region in the top channel, a potential (50 V) was applied at the electrodes and images were captured at emission frequencies of the dyes using appropriate filters cube (described below in fluorescence microscopy). Images were taken every 5 s to obtain data on cell accumulation. After applying 50 V for 3 min, the flow in the side channels with bacteria was stopped and the flow rate of buffer was increased to 200 μL/h. This condition was used for 2 min. This operation resulted in the flow of a fixed amount of bacteria (required for characterization) across the membrane region. At the end, the operational voltage was increased for another 3 min to lyse the accumulated cell population.

### 4.3. Bacterial Sample Preparation

K-12 *E. coli* strain derivative (MG1655) and Green Fluorescence Protein (GFP) expressed *E. coli* (MG1655) were used for different experiments. *E. coli* was inoculated by adding a single colony from pre-made bacteria agar plate to 4 mL of the freshly prepared Luria Broth (LB). In case of GFP expressed *E. coli*, kanamycin (50 μg/mL) was used in the medium. The culture was incubated in a shaker bath for 16 h at 37 °C. Concentration of the bacteria was around 10^8^–10^9^ Colony Forming Units (CFU)/mL after harvesting [53].

Subsequently, 1 mL of culture was transferred to a micro centrifuge tube and centrifuged at 7000 rpm for 7 min. The supernatant was removed by using a pipette and the resulting pellet was washed by using phosphate buffer (1.35 mM KH_2_PO_4_, 2mM Na_2_HPO_4_, 0.05% Tween20, pH 7) 3 or 4 times. Serial dilution was done afterwards to obtain a cell concentration of about 10^6^–10^7^ CFU/mL. Tween20 was used in order to reduce the absorption of cells and debris into the wall of the channels [42,54].

1.5 μL of Syto 9 and 1.5 μL of PI were added to sample when *E. coli* strain K-12 (MG1655) without GFP was used and incubated for 30 min in a dark environment.

### 4.4. Fluorescence Microscopy

A fluorescence microscope was used to monitor all experiments. Two different kinds of fluorescence microscopes, namely Widefield Deconvolution and LumaScope, were used. Widefield Deconvolution (Leica DMI 6000 B, Leica, Solms, Germany) was used in experiments that required simultaneous imaging of Syto 9 and propidium iodide. The images were taken using a charge coupled device (CCD) camera (Hamamatsu Orca ER-AG, Hamamatsu Photonics, Hamamatsu, Japan). LumaScope (LS-500: BF/FL, BIOIMAGER Inc., Concord, ON, USA) was used to image accumulation of GFP expressing bacterial cells.

### 4.5. Plate Counts

The conventional method that is commonly used to check viability and to count the number of cells is the plate counting method. This is a standard method and colonies of bacteria are formed in agar plate. If the bacteria are dead, no colonies form. Thus, we used this method to find out the efficiency of the device. Cell suspensions were diluted with phosphate buffer (pH 7.0) and 100 μL portion of appropriate dilution was spread out in a premade agar plate. After incubation overnight at 37 °C, the colonies from plates were counted.

### 4.6. Quantitative PCR (qPCR) Analysis

Quantitative PCR (qPCR) have been used in order to quantify the amount of DNA after lysis. Primers for use with qPCR were synthesized by standard phosphoramidite chemistry (Integrated DNA Technologies, Coralville, IA, USA). The primers were designed to amplify a 90 bp non-coding stretch of DNA between *degQ* and *degS* in *Escherichia coli* MG1655. qPCR was conducted with the use of CFX96 Real-Time PCR Detection System (Bio-Rad Laboratories, Hercules, CA, USA) and DNA was detected with the use of EvaGreen DNA stain (Biotium, Fremont, CA, USA) according to manufacturer’s directions. All samples were run in triplicate to obtain *C*_T_ values, which is the number of cycles required to reach a threshold signal level. Concentrations of samples were calculated by comparison to a standard curve created from pure *Escherichia coli* DNA.

## 5. Results and Discussion

The operation of the device consisted of two stages: (1) electrophoretic accumulation and (2) electrical lysis. These were controlled by the setting the applied voltage so that the local electric field close to the pores of the membrane are below the critical threshold for lysis in the case of accumulation and above threshold in the case of lysis.

### 5.1. Electrophoretic Accumulation

**Figure 4:**
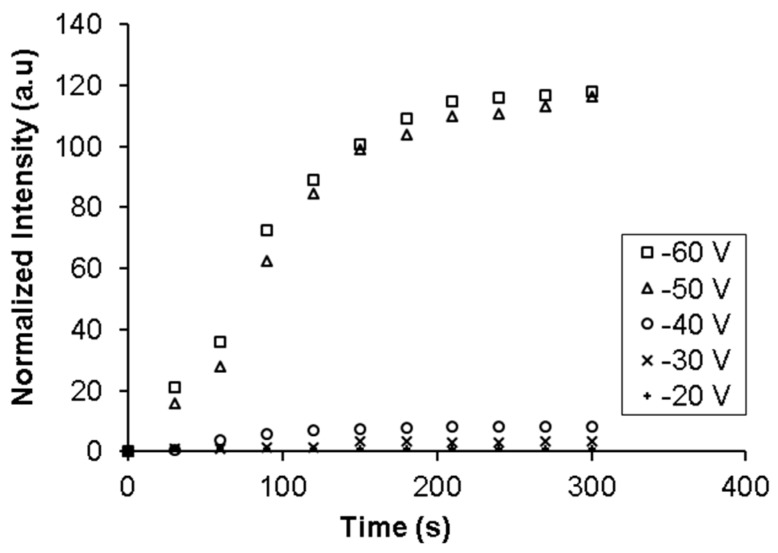
Changes in Syto 9 fluorescent intensity with time due to accumulation of bacteria at the intersection of the sample and the collection channel on the nanoporous membrane at various voltages.

Experiments were conducted to determine the minimum threshold voltage required to accumulate the cells at the nanoporous membrane interface. A flow of 100 μL/h of sample (buffer with concentration of 10^6^–10^7^ CFU/mL of *E. coli*) in the injection channel and 100 μL/h of buffer in the focusing and sample channel was maintained for 5 min. The flows in the focusing and sample channels were used here so that the injected sample volume can be accurately controlled for all the experiments. The cell suspension was stained with Syto 9. Voltages from 20 to 60 V were applied for the entire duration (5 min) when the sample flow was on. The electric field due to the applied voltage was present in the section of the sample channel downstream from the membrane and was in the direction such that it imposed an electrophoretic force on the negatively charged bacteria to move towards the membrane. Fluorescent images of the nanoporous membrane at the intersection of the two channels were taken every 5 s with blue excitation. Results are shown in Figure 4. The fluorescent intensity in the images was quantified using ImageJ software (from NIH).

It can be seen from Figure 4 that the fluorescent intensity at the membrane increased significantly and becomes saturated when the applied voltage is above 50 V. In addition, the intensity does not increase with time when the applied voltage is below 50 V. This observation is expected as movement and accumulation of bacteria in the flowing sample is due to a combination of the electrophoretic force on it pulling it onto the membrane and the drag force of the fluid pulling it towards the outlet. When the applied voltage is lower than 50 V, the electrophoretic force on the bacteria due to the electric field in the channels is not sufficiently high to overcome the drag force of the fluid, which prevents accumulation. As the applied voltage is increased, the electrophoretic force increases while the drag force remains constant. At 50 V the electrophoretic force is strong enough to overcome the drag force and pull the bacteria from the sample onto the membrane. The bacteria rapidly accumulate on the membrane pores until all pores are blocked. Once the pores are blocked, the electric field in the sample channel falls and no more net accumulation takes place. The rate of accumulation and the amount of saturation remains steady even when the applied voltage is increased from 50 and 60 V as the volumetric flow rate of the bacteria across the membrane is the same irrespective of the applied potential and therefore, the rate of accumulation will be similar once the threshold voltage for accumulation has been achieved.

### 5.2. Effect of Sample Concentration on Accumulation

**Figure 5:**
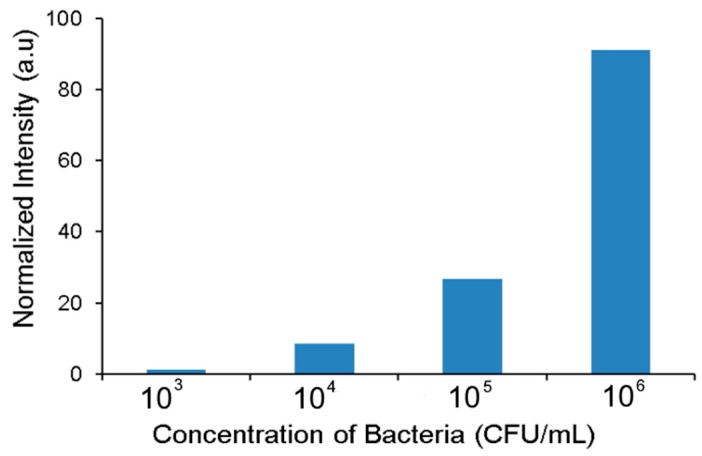
Accumulation at different concentration of cells for 50 V and 20 min.

The purpose of the accumulation step becomes important when the bacterial concentration is low. In order to demonstrate accumulation under various sample concentrations, sample solution containing GFP expressing *E. coli* at concentrations ranging from ~10^2^–10^6^ CFU/mL were tested. The flow conditions were similar to the previous experiment with a sample flow rate of 100 μL/h. A voltage of 50 V was applied for 20 min and images of the nanoporous membrane at the intersection of two channels were taken every 30 s. The intensity of the accumulated bacteria (Figure 5) shows a steep increase with sample concentration.

Significant accumulation was obtained for sample concentration between 10^4^–10^6^ CFU/mL within 20 min. The accumulated concentration was much lower when the sample concentration was 10^3^ CFU/mL. This is as expected as at the set flow rate and duration, only ~20 bacteria would have flowed past the accumulation region at a sample concentration of 10^3^ CFU/mL. In the case of 10^2^ CFU/mL only ~2 bacteria would be present and it did not show any measureable intensity variation under the conditions studied.

### 5.3. Electrical Lysis

Once the threshold voltage (50 V) for accumulation was determined we conducted experiments to determine whether the accumulated cells can be lysed. In this experiment an *E. coli* sample (concentration of 10^6^–10^7^ CFU/mL) stained with Syto 9 and PI was introduced in the injection channel and buffer was introduced from the focusing and sample channels (Figure 1a) with a flow rate of 100 μL/h. A voltage of 50 V was applied for 8 min and images of the nanoporous membrane at the intersection of two channels were taken every 5 s for blue and green excitations.

**Figure 6:**
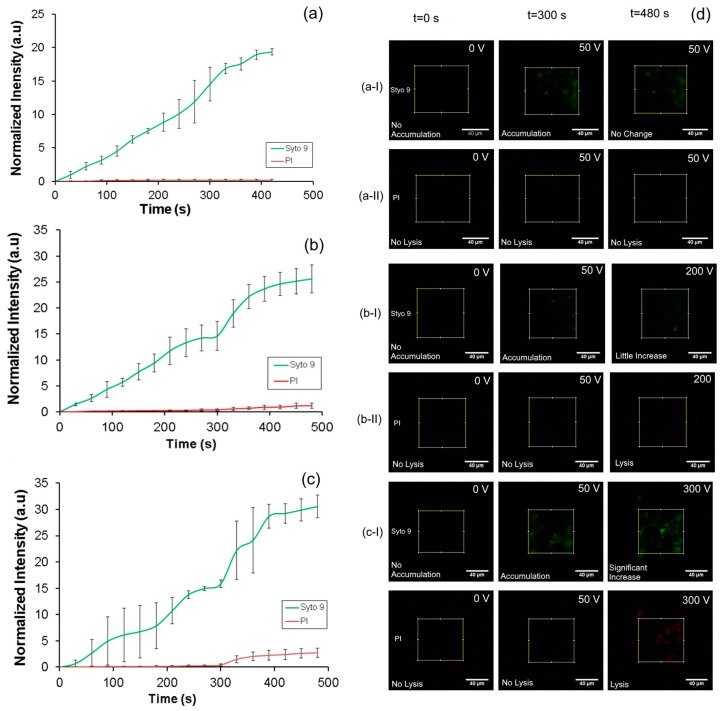
Intensity profile for accumulation followed by lysis when tested with Syto 9 and PI (**a**) 50 V applied for 8 min; (**b**) 50 V for 5 min and 200 V for 3 min; (**c**) 50 V for 5 min and 300 V for 3 min; (**d**) Time sequenced fluorescent images (Syto 9 (I) and PI (II)) at the intersection of the microchannels, (**a**) when the applied voltage was 50 V; (**b**) when the applied voltage was 50 V for 5 min and then increased to 200 V for 3 min; (**c**) when the applied voltage was 50 V for 5 min and then increased to 300 V for 3 min. scale bar = 40 μm.

The intensity of fluorescence from Syto 9 and PI over 8 min of accumulation at 50 V is shown in Figure 6a. Images of the nanoporous membrane interface for Syto 9 and PI at various time points during accumulation (*t* = 0, *t* = 300 s and *t* = 480 s) during application of 50 V are shown in Figure 6d((a-I),(a-II)). These results show that a rapid increase in the Syto 9 intensity over time whiles the intensity of PI did not increase indicating that the accumulating bacteria are intact and not lysed. At the applied voltage of 50 V, the electric field in the nanopores was 308 V/cm and in the main sample channel was 62.11 V/cm, both of which are well below the critical electric field required to lyse the cell. Modelling using COMSOL multiphysics (version 4.3, COMSOL, Inc., Burlington, MA, USA) showed that the intensity of the electric field dropped quickly from the nanopores into the bulk solution, but was ~80% at 10 µm into the bulk solution. This indicates that the electric field in the pores is a good measure of the intensity that the bacteria that have been pulled to the nanopores are experiencing.

After confirming accumulation of intact cells, we conducted experiments to demonstrate cell lysis using higher voltages as discussed in experimental setup and procedure section. In these experiments, accumulation was done for 5 min using a procedure similar to the one described before. Subsequently, the flow in the injection channel and buffer channels were stopped and the flow in the sample channel (200 µL/h) was continued in order to flush away the remaining sample solution in the upstream region. Then the applied potential was increased to 200 and 300 V which corresponds to local electric field at the nanopores of 1230 V/cm (≈2 µA) and 1860 V/cm (≈3 µA) respectively.

The intensity profile of both Syto 9 and PI at the membrane interface as presented in Figure 6b shows rapid accumulation of bacteria till 5 min when steady state is reached. Subsequently, when the higher voltage is applied at 300 s, there was a rapid increase in the intensity of both Syto 9 and PI that also subsequently saturated with time. This behavior is as expected. A continuous flow of cell for the first 3 min led to the gradual increase in accumulation of the bacteria which is indicated by the increase in the intensity of Syto 9. Subsequently, the flow of the sample was stopped and only the buffer flow was maintained in the next 2 min, leading to a steady state in the amount of bacteria accumulated. This procedure was done in order to precisely define the amount of bacteria injected into the device. During 5 min the intensity of PI did not increase indicating that bacterial lysis did not occur.

Subsequently, when the applied potential was increased to 200 V and applied for 3 min the cells lysed leading to a sharp increase in fluorescence intensity of Syto 9 (Figure 6d(b-I)). These are attributed to the higher permeability of the lysed membrane as compared to the intact membrane to Syto 9. More significantly, the intensity of the PI (Figure 6d(b-II)) also increased, providing a clear indication that the cells are lysed at this applied potential. The electric field calculated at the nanopores was 1230 V/cm at an applied potential of 200 V which was higher than the threshold for lysis. No lysis was observed in the microchannels as the electric field was around 250 V/cm and well below the threshold. A similar trend in the fluorescence intensities for Syto 9 and propidium iodide was also observed when 300 V (Figure 6c) was applied during the lysis phase after accumulation at 50 V for 5 min.

One of the significant benefits of the current design is that it reduces the effect of joule heating on the lysis due to continuous flow of the sample/buffer during the entire accumulation and lysis process. For instance, at the maximum applied voltage of 300 V, a current of ~3 µA was measured indicating a power dissipation of 0.9 mW over the entire device. Assuming that all this energy is consumed in increasing the temperature of the liquid and is not dissipated through the walls, a temperature increase of ~4 °C can be calculated. The real temperature increase is likely to be smaller and demonstrates that lysis is not affected by Joule heating.

### 5.4. Effect of Flow Rate

**Figure 7:**
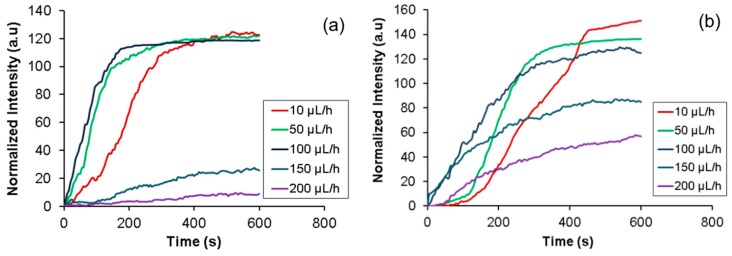
Characterization of flow rate for operational voltage of (**a**) 50 V and (**b**) 100 V.

Determination of the influence of flow rate on the collection efficiency during the accumulation phase is critical for the efficient functioning of this device. The drag force imposed by pressure driven flow moves the bacteria away from the membrane while the electrophoretic force pulls it on to the membrane. A high flow rate is desirable in order to process a large volume of sample. However, high flow rates will require higher applied voltage in order to overcome the drag force and accumulate the bacteria onto the membrane. The applied voltage cannot be increased beyond the point where the threshold electric fields are reached either at the nanopores, or more importantly, at the channel as it would lyse the bacteria. Therefore, experiments were conducted in order to determine the optimal flow rates to operate this device in the accumulation mode at applied voltages of 50 and 100 V (below the threshold for lysis). GFP expressed *E. coli* with a concentration of 10^6^–10^7^ CFU/mL was used for this experiment and the set-up was same as discussed in experimental section. The experiment was conducted for 10 min with the sample and buffer flowing for the entire experiment. Five different flow rates (10, 50, 100, 150, and 200 μL/h) were used. The results are shown in Figure 7a, b for an operational voltage of 50 V and 100 V respectively.

When the applied voltage was 50 V (308 V/cm) (Figure 7a), the bacteria accumulate on the membrane at flow rates at or below 100 µL/h, while the accumulation is significantly reduced above it. This indicates that the electrophoretic force on the bacteria due to the electric field of 308 V/cm was sufficient to overcome the drag force due to a flow of 100 µL/h or below. The rate of accumulation at low flow rates was also found to increase with the flow rate. This is as expected since the amount of bacteria passing the membrane is higher for higher flow rates. Finally, it can be seen that the accumulation saturates the membrane after a certain time and equilibrium is reached.

When the applied voltage was increased to 100 V (616 V/cm) (Figure 7b), the distinction between high and low flow rates are not as dramatic. Since the electrophoretic force is stronger, bacteria accumulate on the membrane even at high flow rates. However, the total accumulated amount at equilibrium is found to be inversely proportional to the flow rate. The trend for the rate of accumulation was similar to the one observed at 50 V. The rate was slower possibly due to lysis of some of the bacteria accumulated at the higher potential. From these experiments, it was concluded that 100 μL/h was the optimal flow rate for rapidly accumulating the cells at the nanoporous interface.

### 5.5. Characterization of Co-Flow and Cross-Flow

**Figure 8:**
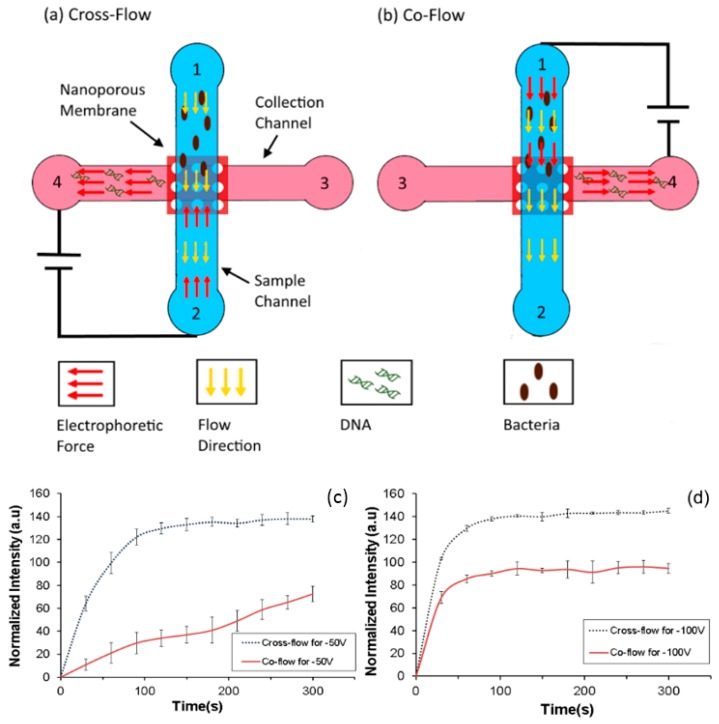
Device configuration for (**a**) cross-flow and (**b**) co-flow. Characterization of direction of Electric field for operational voltage of (**c**) 50 V and (**d**) 100 V.

Various configurations of flow can be used in the accumulation device. The flow of the sample and the direction of the electric field could be the same (co-flow) or opposite (cross-flow) to the pressure driven flow in the main sample channel. It is expected that when the device is operated in the cross-flow configuration the cells have a higher residence time above the nanoporous interface and therefore are likely to accumulate more as compared to the co-flow configuration. The devices used for characterizing the effect of flow configurations are shown in (Figure 8a,b). Similar to the previous devices, it consists of two channels—a sample (top) and a collection (bottom) channels with a nanoporous polycarbonate membrane sandwiched between them.

In both the configurations, the sample (10^6^–10^7^ CFU/mL) was flowed from reservoir 1 and buffer from reservoir 3 with a flow rate of 100 μL/h and the applied voltage was 50 V and 100 V. The dimensions of the device (80 µm × 50 µm × 10 mm) were such that the intensity of the electric field was the same in both the configurations while the direction changed. Accumulation of bacteria was compared by measuring the intensity of GFP from GFP expressing *E. coli* at the intersection for both setups over duration of 5 min.

As seen in Figure 8c,d, the accumulation in the cross flow configuration is higher and faster than that for co-flow configuration at both the accumulating voltages as expected. It can also be seen that at 100 V a larger amount of bacteria can be accumulated as compared to 50 V in the co-flow configuration. In the cross flow configuration, the direction of electrophoretic force is opposite to the flow. This increases the residence time of the bacteria under the influence of the electric field. The increased accumulation is due to higher residence time of the bacteria under the influence of the electric field in the channel in the cross flow configuration as compared to the co flow configuration.

**Figure 9:**
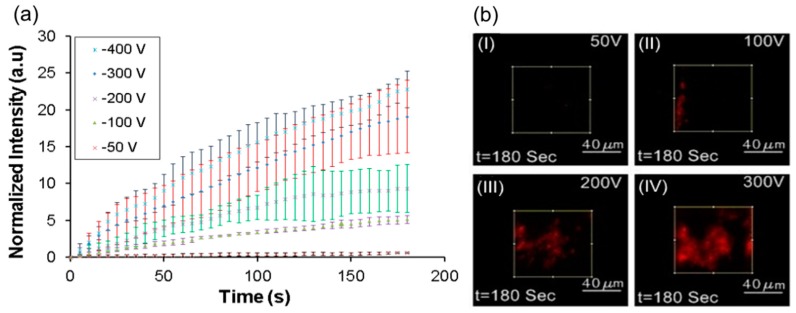
(**a**) Characterization of applied voltage for cell Lysis; (**b**) images at intersection after 3 min for different operational voltage when cells are stained with PI. Scale bars are 40 μm. (I) 50 V, (II) 100 V, (III) 200 V and (IV) 300 V.

The applied voltage and the resultant electric field at the nanopores is the most important parameter determining the efficiency of the lysis process. In order to determine the effect of voltage, *E. coli* sample (10^6^–10^7^ bacterial cells/mL) , incubated with PI for 30 min, was flowed through the device at 100 μL/h in a cross-flow configuration for a duration of 3 min while being exposed to various applied voltages of 50, 100, 200, and 300 V. It should be noted that in this configuration, the cells are simultaneously captured on the membrane and lysed immediately. The intensity of the PI at the nanoporous membrane which is indicative of cell lysis is plotted in Figure 9a for various applied voltages. Images of the membrane interface at various applied voltages at the same instance of time are shown in Figure 9b.

It can be seen in Figure 9b(I) that lysis is minimal at 50 V applied voltage, as the electric field (308 V/cm) at the nanoporous interface was much lower than the threshold value for lysis. When the voltage was increased to 100 V (electric field of 616 V/cm at nanopores) some of the cells lyse (Figure 9b(II). It has been reported in the literature that cell lysis starts at 600 V/cm for Chinese hamster ovary (CHO) cells [55] and that the lysis also depends upon the duration of field [49]. Although bacterial cells used here are quite small compared to CHO cells, the exposure time was much higher. The higher exposure time close to the threshold for lysis could have led to the lysis of some cells at low efficiency. When the applied voltage was increased to 200 V (electric field 1232 V/cm) or 300 V, a significant increase in fluorescence intensity of PI was observed at the nanoporous interface (Figure 9b(III,IV)) indicating significant increase in cell lysis. At 300 V, where the electric field was around 1860 V/cm, the entire interface was saturated by red fluorescence and cells were completely disintegrated. It can be concluded that increasing the voltage leads to higher capture and lysis of bacteria from the sample.

### 5.6. Lysis Efficiency

**Figure 10:**
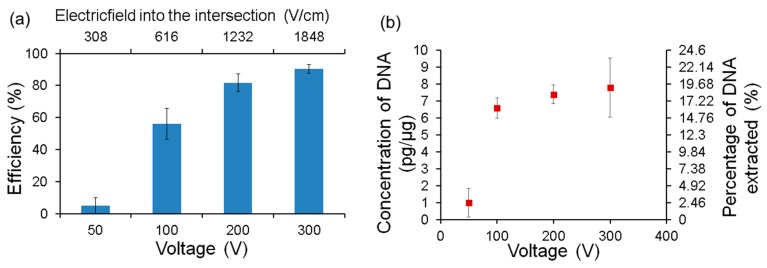
(**a**) Lysis efficiency of the device. Mean value and error bars were obtained from multiple runs (3 runs); (**b**) concentration of DNA extracted from collection channel.

Fluorescent measurements provide only qualitative understanding of the lysis phenomena. In order to get a quantitative estimation, separate experiments were conducted and plate counting of bacterial culture was done to determine the lysis efficiency. After running the test as mentioned in the previous section, 10 μL of solution was collected from sample channel and collection channel outlet. After collection, the sample was diluted and plated on an agar plate. First, the test was run without applying any potential and was used as control. Efficiency was then calculated by comparing to this control. The results are shown in Figure 10a.

They show that the efficiency of lysis increases with the applied voltage. It is 56% at 100 V, 82% at 200 V (1232 V/cm) and 90% at 300 V (1860 V/cm) while only 5% were lysed at 50 V. This result confirms and quantifies the trend observed using fluorescence measurements. In addition, no colonies were formed from the fluid collected from the collection channel confirming that intact bacteria did not pass through the membrane.

To confirm and quantify the genomic DNA after lysis, quantitative polymerase chain reaction (q-PCR) was also performed. *E. coli* with a concentration of 10^8^ CFU/mL was flowed from the injection channel at a flow rate of 100 µL/h and buffer was supplied from main and focusing channels with same flow rate. After running the test for 5 min, the sample was collected from the collection channel and qPCR analysis was performed using a pair of primers targeting *E. coli* DNA. It can be seen from Figure 10b that the amount of DNA collected in the collection channel is significantly higher when the applied voltage is higher than 100 V. In addition, we calculated the total weight of DNA that is present in the cells in the sample from its concentration and estimated the yield of the device. Almost 20% of the DNA is extracted from the sample when the applied voltages are 200 and 300 V as shown in Figure 10a. Although this is not a measure that is usually reported in other publications, it is useful in determining the efficacy of the device in the field. The low yield could be due to adherence of the DNA in the cell debris or on the internal surfaces of the devices itself. Further work is needed to visualize, quantify and reduce it in order to improve the yield.

## 6. Conclusions

We have successfully fabricated a simple and rapid cell lysis system by using low voltage and tested it for *E. coli* cells. The novelty of the device is the integration of trapping and lysis. We have demonstrated that there is a threshold electric field below which the device operates predominantly as an accumulator of cells suspended in the microchannel and above which it starts to lyse the accumulated cells. For instance, application of 50 V (electric field 308 V/cm) to the device with a flow rate in the channel of 100 μL/h has led to rapid accumulation of cells on top of the membrane. Subsequent to the accumulation when normal *E. coli* with propidium iodide has been used, application of 300 V (electric field 1850 V/cm) has led to the change in color to red indicating that the cells have been lysed. Characterization of flow rate, voltage and direction of electric field has been done to determine optimal operating parameters. Using the best condition 90% of bacterial cells have been lysed. We are presently trying to embed electrodes very near to the membrane, so that we can reduce the operational voltage for lysis as well as integrate the DNA amplification with this lysis.

## Figures and Tables

**Table 1:** Relation between pore sizes and operational voltage to generate the required voltage for cell lysing.

Pore Size (μm)	Pore Density (Pore/cm^2^)	Thickness of the Membrane (μm)	Number of Pores at the 80 µm × 80 µm Intersection	Required Voltage (Approx.) to Generate 1600 V/cm (V)
5	4 × 10^5^	10	26	165
1	2 × 10^7^	11	1280	325
0.4	1 × 10^8^	10	6400	260
0.2	3 × 10^8^	10	19,200	195
0.1	4 × 10^8^	6	25,600	66
0.08	4 × 10^8^	6	25,600	35
